# “Downhill” Esophageal Varices due to Dialysis Catheter-Induced Superior Vena Caval Occlusion: A Rare Cause of Upper Gastrointestinal Bleeding

**DOI:** 10.1155/2013/830796

**Published:** 2013-02-20

**Authors:** Suresh Kumar Nayudu, Anil Dev, Kalyan Kanneganti

**Affiliations:** ^1^Division of Gastroenterology and Hepatology, Bronx Lebanon Hospital Center, Albert Einstein College of Medicine, Yeshiva University, Bronx, NY, USA; ^2^Department of Medicine, Bronx Lebanon Hospital Center, Albert Einstein College of Medicine, Yeshiva University, Bronx, NY, USA

## Abstract

“Downhill” varices are a rare cause of acute upper gastrointestinal bleeding. Rarely these varices are reported in patients receiving hemodialysis as a complication of chronic dialysis vascular access. We present a case of acute upper gastrointestinal bleeding in an individual with end-stage renal disease receiving hemodialysis. Esophagogastroduodenoscopy revealed “downhill” varices in the upper third of the esophagus without any active bleeding at the time of the procedure. An angiogram was performed disclosing superior vena caval occlusion, which was treated with balloon angioplasty. Gastroenterologists should have a high index of suspicion for these rare “downhill” varices when dealing with acute upper gastrointestinal bleeding in patients receiving hemodialysis and manage it appropriately using endoscopic, radiological, and surgical interventions.

## 1. Introduction

“Downhill” varices are a rare cause of acute upper gastrointestinal bleeding. These varices have been reported in association with superior vena caval (SVC) obstruction secondary to extrinsic compression from tumors or venous thrombosis. Very rarely the SVC obstruction has been reported in literature in individuals with end-stage renal disease (ESRD) as a complication of chronic dialysis access [1–8]. We present a case of acute upper gastrointestinal bleeding in an individual with ESRD receiving hemodialysis. He previously underwent multiple angioplasties due to malfunctioning dialysis access resulting in SVC occlusion. 

## 2. Case Presentation

A 48-year-old African American man presented to our emergency department (ED) with hematemesis and melena. Associated symptoms included dizziness and generalized weakness. He denied any abdominal pain or previous episodes of gastrointestinal bleeding. He also denied prior endoscopic work-up. His medical history included ESRD on maintenance hemodialysis, seizure disorder, dyslipidemia, and hypertension. His surgical history included repair of abdominal aortic aneurysm, aortic valve replacement, and construction of arteriovenous (AV) fistula multiple times for dialysis access. Patient was receiving Coumadin for his aortic valve replacement; however, patient was noncompliant with Coumadin for few days prior to his admission. He denied the use of tobacco, alcohol, or recreational drugs.

Physical examination on the patient revealed hypotension with systolic blood pressure of 80 millimeters of mercury. Patient was in mild distress with clear mentation and was able to provide clear history. His abdominal examination was normal with no distension or tenderness. Rectal examination revealed black tarry stool. Examination of his upper extremities revealed dilated, tortuous veins at the site of previous AV fistulas without any evidence of bleeding. Initial laboratory tests revealed hemoglobin of 6 grams/dL with normal platelet count and coagulation profile. His blood urea nitrogen and serum creatinine were elevated consistent with his underlying renal disease. His liver chemistries were within normal limits. 

Review of his old medical records revealed that he underwent angioplasties of cephalic vein, subclavian vein, and superior vena cava multiple times for failed dialysis access. He also underwent AV fistula construction of bilateral upper extremities with attempted revision and repair several times. Temporary hemodialysis access catheter placement through subclavian vein was also done multiple times. 

**Figure 1:**
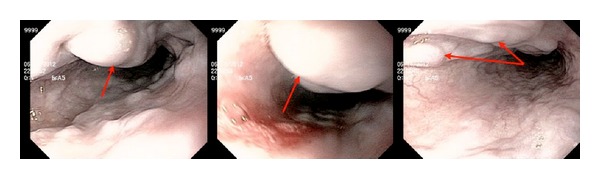
Esophagogastroduodenoscopy showing “downhill” varices in the upper 3rd of esophagus.

After adequate resuscitation with packed red blood cells (PRBC) and intubation for airway protection, an emergent esophagogastroduodenoscopy was performed. Endoscopy revealed grade III esophageal varices (Figure 1) in the upper third of the esophagus. No active bleeding was noted from esophageal varices at the time of the procedure; however, altered blood was noted in the stomach. His duodenum was grossly normal and no other bleeding lesions were found either in the stomach or in the duodenum. Due to the absence of active bleeding, no endoscopic intervention was performed. Subsequently, the patient was transferred to medical intensive care unit (MICU) for supportive care and close monitoring.

Given the patient's history of multiple dialysis access failures, an angiogram was performed by interventional radiology. The angiogram revealed complete occlusion of the superior vena cava (SVC). Recanalization and angioplasty of SVC were performed with 6- and 10-millimeter balloons with satisfactory results. 

Subsequently his general clinical condition improved and he was weaned from mechanical ventilation. His previously noted dilated, tortuous veins of his bilateral upper extremities collapsed after the angioplasty. Hemoglobin and hematocrit improved with transfusions and the patient was transferred to the medical floor. The patient's condition remained stable and he was discharged a few days later. He remained asymptomatic with stable hemoglobin on his follow-up visit in the gastroenterology clinic. 

## 3. Discussion

“Downhill” varices were first described by Felson and Lessure in 1964 [9], followed by several case reports. They are seen in patients without portal hypertension in contrary to the “uphill” varices [10, 11]. They are dilated veins resulting from SVC obstruction whose blood flow is directed caudally towards azygous vein [12] or inferior vena cava (IVC). They are either located in the upper esophagus or may involve the entire esophagus depending on the level of obstruction above or below azygous venous system, respectively [13–15]. Various neoplastic lesions involving neck and thorax have been reported to cause SVC compression leading to “downhill” varices [16]. There have been also reports of “downhill” varices in patients with systemic vasculitis syndromes like Behcet's disease [17].

In few instances, “downhill” varices have been reported due to iatrogenic causes like pacemaker insertion [18] and hemodialysis access [1–8]. Acute upper gastrointestinal bleeding is the commonest presentation of these “downhill” varices, where the etiology is an iatrogenic cause such as recurrent hemodialysis vascular access manipulation leading to SVC stenosis. 

There are no definitive recommendations on how to screen and manage “downhill” varices. Less invasive interventions like percutaneous radiological SVC angioplasty with stent placement have been tried with success [4, 7]. Endoscopic banding and sclerotherapy have been also attempted with limited success [19, 20]. Surgical modality has been successful in cases where SVC obstruction was secondary to extrinsic compression. Surgical removal of the tumor causing the extrinsic compression in these cases resulted in relieving obstruction there by leading to therapeutic benefit from “downhill” varices [12].

This case represents a rare but physiologically plausible cause of acute upper gastrointestinal bleeding in individuals with recurrent dialysis access failures leading to SVC thrombosis. Gastroenterologists must show a high index of suspicion and awareness of the patient's overall clinical condition in dealing with acute upper gastrointestinal bleeding in hemodialysis patients. Though no standard recommendations guiding physicians to manage acute bleeding associated with “downhill” varices are available [21], we suggest that awareness, prompt diagnosis, and management on a case by case basis using available endoscopic, radiological, and surgical interventions can be successful.
